# Comparing the efficacy and safety of unilateral versus bilateral spinal anesthesia: a meta-analysis and systematic review

**DOI:** 10.1080/07853890.2026.2689575

**Published:** 2026-06-18

**Authors:** Ji-Xiang Wan, Si-Si Zeng, Jia-Man Li, Yuan Wang, Jia-Bei Li, Fang-Jun Wang

**Affiliations:** a Department of Anesthesiology, West China Hospital of Sichuan University Ziyang Hospital, Ziyang Central Hospital, Ziyang City, Sichuan Province, China; b Affiliated Hospital of North Sichuan Medical College, Nanchong City, China; c Xinqiao Hospital of Chongqing, Second Affiliated Hospital of Army Medical University, Chongqing, China

**Keywords:** Bilateral, meta-analysis, spinal anesthesia, systematic review, unilateral

## Abstract

**Background:**

Unilateral spinal anesthesia has gained increasing attention in recent years. Emerging evidence suggests that it provides comparable analgesia to conventional bilateral spinal anesthesia while reducing adverse effects, and its efficacy and safety compared to bilateral spinal anesthesia remains controversial.

**Objective:**

This systematic review and meta-analysis aims to evaluate and compare the efficacy and safety of unilateral versus bilateral spinal anesthesia.

**Design:**

Systematic reviews and meta-analysis of randomized controlled trials (RCTs).

**Data sources:**

A systematic search was conducted across PubMed, EMBASE, and Cochrane Library from inception to December 10, 2024.

**Eligibility criteria:**

Included studies were randomized controlled trials involving adult patients (≥18 years) undergoing surgery under spinal anesthesia, comparing unilateral versus bilateral spinal anesthesia for efficacy and adverse effects. Studies that focused exclusively on either unilateral or bilateral spinal anesthesia were excluded. The comparator group used the same local anesthetic as the experimental group, with no restrictions on adjuncts (e.g. fentanyl, morphine).

**Results:**

Nineteen randomized controlled trials including 1191 patients met the inclusion criteria. Compared with bilateral spinal anesthesia, unilateral spinal anesthesia has a longer onset of sensory blockade (MD = 2.58, 95% *CI*: 0.93 to 4.22, *p* = 0.002), a shorter duration of sensory blockade (MD = −27.83, 95% *CI*: −39.25 to −16.42, *p* < 0.00001). In addition, unilateral spinal anesthesia significantly reduced the incidence of hypotension (RR = 0.40, 95% *CI*: 0.31 to 0.52, *p* < 0.0001), nausea and vomiting (RR = 0.20, 95% *CI*: 0.07 to 0.56, *p* = 0.002), and post-dural puncture headache (RR = 0.44, 95% *CI*: 0.23 to 0.81, *p* = 0.009). No statistically significant differences were observed in bradycardia and urinary retention. Collectively, these findings support unilateral spinal anesthesia as a strategy that may enhance perioperative safety while maintaining adequate anesthetic efficacy in appropriately selected patients

**Conclusions:**

Unilateral spinal anesthesia may offer a favorable balance between anesthetic efficacy and safety compared with bilateral spinal anesthesia, although its clinical utility may depend on surgical duration and patient characteristics.

## KEY POINTS

1. Unilateral spinal anesthesia is associated with a significantly lower incidence of adverse effects, including hypotension, nausea, vomiting, and headache.
2. Bilateral spinal anesthesia provides faster onset and longer duration of sensory blockade but carries a higher risk of complications.
3. The dose of local anesthetic plays a critical role in the efficacy and duration of unilateral spinal anesthesia.

## Introduction

Spinal anesthesia is a widely used regional anesthesia technique, known for its rapid onset, reliable sensory and motor blockade, and quick recovery [1]. While bilateral spinal anesthesia is the conventional approach, delivering uniform blockade to both sides of the body, its association with significant hypotension—especially in elderly patients [2,3] and those at high risk (Elderly individuals and patients with cardiovascular disease) [4]—has prompted exploration of alternatives like unilateral spinal anesthesia [5,6].

Unilateral spinal anesthesia, which targets one side of the spinal column, aims to achieve localized blockade [7,8]. Unilateral spinal anesthesia is typically achieved by administering hyperbaric or hypobaric local anesthetic with the patient in the lateral decubitus position. Maintaining this position for 10–20 min promotes preferential distribution of the anesthetic to the operative side, resulting in asymmetric sensory and motor blockade [5,8,9]. The technique is most commonly used in unilateral lower-limb orthopedic procedures, ambulatory surgeries, and other infraumbilical operations in which unilateral block is sufficient. Compared with conventional bilateral spinal anesthesia, unilateral techniques often employ lower doses of local anesthetics, which may limit the extent of sympathetic blockade and contribute to improved hemodynamic stability [6,10]. By enhancing hemodynamic stability, it offers a safer alternative for patients at elevated cardiovascular risk. Unilateral spinal anesthesia has garnered increasing attention in recent years. Several studies have demonstrated that it provides comparable analgesia with fewer adverse effects than bilateral approaches [5,11]. However, other reports have highlighted potential drawbacks, including prolonged recovery times and incomplete sensory or motor block [12,13]. Such inconsistencies emphasize the need for a comprehensive review.

This systematic review and meta-analysis is designed to evaluate the efficacy and safety of unilateral versus bilateral spinal anesthesia. By synthesizing current evidence, we seek to clarify the strengths and limitations of each approach to inform clinical practice.

## Methods

This systematic review adhered to the PRISMA guidelines and current Cochrane Collaboration recommendations. The protocol was registered in the International Prospective Register of Systematic Reviews (Registration Number: CRD42024625887; available at: https://www.crd.york.ac.uk/prospero/#myprospero).

### Search strategy

A systematic search of PubMed, EMBASE, and the Cochrane Library was conducted from database inception to December 10, 2024. The search strategy included the terms ‘unilateral spinal anesthesia’ and ‘bilateral spinal anesthesia.’ Only randomized controlled trials were eligible for inclusion. Additionally, the U.S. Clinical Trials Registry was thoroughly hand-searched for in-progress, terminated, or completed studies with available results that met inclusion criteria. The complete search strategy for one database is available in Supplementary Appendix 1.

### Eligibility criteria

Inclusion criteria are as follows: (1) published in English; (2) enrolled adult patients (≥18 years) undergoing outpatient day surgery or inpatient surgery under spinal anesthesia; (3) directly compared unilateral and bilateral spinal anesthesia within the same study; (4) The control and experimental groups must use the same local anesthetic.

Exclusion criteria: (1) Studies evaluating only unilateral or only bilateral spinal anesthesia without a direct comparative group; (2) Studies not published in full; (3) Review articles; (4) Case reports; (5) Articles published in non–peer-reviewed journals.

### Study selection

Two reviewers (J.X.W. and S.S.Z.) independently screened the results produced by the search strategy from the selected databases. All potentially eligible citations, based on title and abstract screening alone, had their full-text versions retrieved for a thorough re-evaluation of inclusion criteria. Any disagreements on full-text study inclusion were discussed until a consensus was reached. If a consensus could not be achieved after discussion, a third reviewer (F.J.W.) was consulted to make the final decision.

### Data extraction

Two authors (X.J.W. and S.S.Z.) independently extracted data into a pre-designed spreadsheet. Discrepancies were resolved through re-examination or consultation with a third author (F.J.W.). If necessary, we will contact the original author within permissible limits to provide relevant experimental data or publicly available data. If the author cannot be reached, an explanation will be provided. A third reviewer (J.M.L) cross-checked extracted values against source manuscripts. Extracted information included the year of publication, type of surgery performed, local anesthetic dose and concentration, primary outcomes (onset and duration of anesthesia), and secondary outcomes (incidence of adverse effects: hypotension, bradycardia, nausea, vomiting, urinary retention, headache).

### Primary outcomes

 

1. Onset time of anesthesia: The onset time of anesthesia was further classified into sensory and motor block onset times. Sensory block onset time was defined as the interval between intrathecal administration of the local anesthetic and achievement of a predefined sensory block level. Motor block onset time was defined as the interval from anesthetic administration to achieving a modified Bromage score of ≥2 on the operative side (0 = no paralysis, able to flex knee and ankle; 1 = unable to raise extended leg but able to flex knee; 2 = unable to flex knee but able to flex ankle; 3 = unable to move the lower limb).
2. Duration of anesthesia: The duration of anesthesia was further categorized into sensory and motor block durations. Sensory block duration was defined as the time from the performance of spinal anesthesia to regression of sensory block to a predefined level, while motor block duration was defined as the time from the performance of spinal anesthesia to regression of motor block to a predefined level.

### Secondary outcomes

 

1. Hypotension (The definition of hypotension in each primary study was adopted as the definition of hypotension in this study).
2. Bradycardia.
3. Urine retention.
4. Nausea and vomiting.
5. Post dural puncture headache.

### Risk of bias assessment

Two reviewers (J.X.W. and Y.W.) assessed the risk of bias using the Cochrane Collaboration tool [14], evaluating random sequence generation, allocation concealment, blinding, incomplete outcome data, selective reporting, and other biases. Disagreements were resolved through discussion and, if needed, by consulting a third reviewer (F.J.W.). For analyses with more than 10 studies, publication bias was assessed using Egger’s test in Stata software [15]. Evidence strength was evaluated using GRADE guidelines [16,17] and categorized as high, moderate, low, or very low, with results summarized in Supplementary Data Table 1.

### Statistical analysis

Statistical analyses were performed using RevMan (version 5.4.1, Cochrane Collaborative, 2020). For outcomes reported as medians with interquartile ranges, means and standard deviations were calculated using a validated imputation method before meta-analysis. Descriptive statistics were calculated to determine the mean difference (MD) using the random-effects inverse variance method, with results expressed as MD and 95% confidence intervals (CIs). A two-tailed *p* value of <0.05 was considered statistically significant.

For dichotomous outcomes, meta-analyses were conducted using risk ratio (RR) and the Mantel–Haenszel random-effects method, with results reported as RR and 95% CI. Statistical significance was defined as a *p* value of <0.05.

Heterogeneity was assessed with the I^2^ test, where I^2^ > 40% indicated significant heterogeneity. Subgroup analyses were conducted to explore heterogeneity in primary outcomes based on local anesthetic doses. In the same-dose subgroup, consistent doses of local anesthetics were used in both unilateral and bilateral spinal anesthesia groups. Conversely, in the different-dose subgroup, lower doses were administered to the unilateral group compared to the bilateral group. Sensitivity analyses were performed by sequentially excluding individual trials to assess their impact on the pooled RR.

## Results

### Results of the literature search

**Figure 1.**
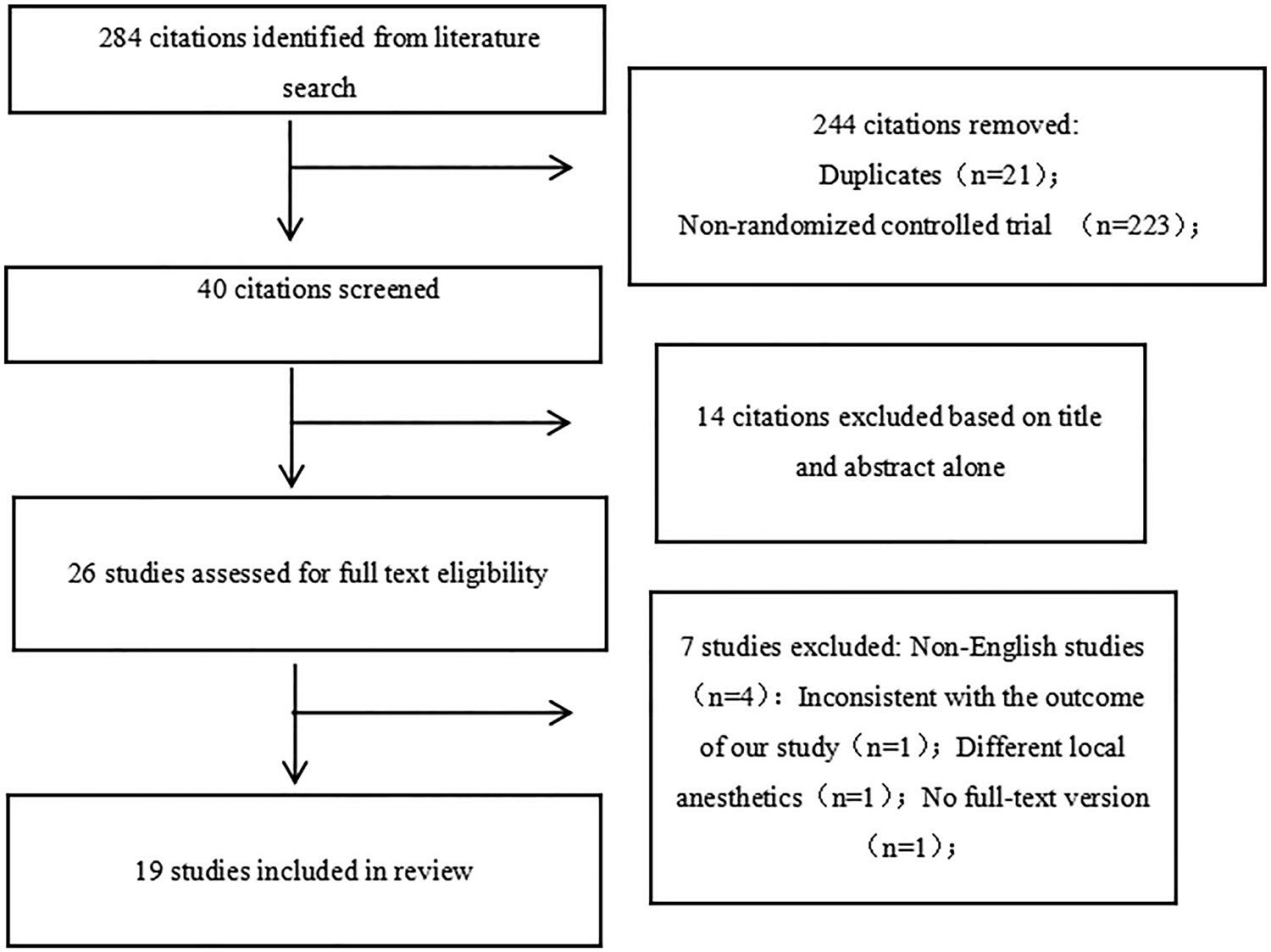
Study flow diagram.

The systematic search identified 284 citations. After removing duplicates, a total of 263 citations were screened, with 223 excluded for not being randomized controlled trials (RCTs). Fourteen citations were excluded following the title and abstract review. One study[18] was excluded due to unavailable full text, four studies [19–22] were excluded because they were published in languages other than English, one [23] was excluded for inconsistent outcomes (This study only recorded continuous changes in blood pressure, differing from the outcomes recorded in other studies), and one [24] for using different local anesthetics in the control and experimental groups. Ultimately, 19 studies met the inclusion criteria and were included in the meta-analysis (Figure 1).

### Study characteristics

The characteristics of the included RCTs are summarized in Table 1.

**Table 1.** Characteristics of the included studies.

Author, Year	Groups (n)	Surgery	Drugs and dosages		Intervertebral space
			Unilateral	Bilateral	
A. CASATI 1998[6]	1.USA (15); 2.BSA (15)	Elective unilateral lower limb orthopedic surgery	8 mg of 0.5% hyperbaric bupivacaine	15 mg of 0.5% hyperbaric bupivacaine	L3-4
A. Casati 1999[25]	1.USA (59); 2.BSA (58)	Elective orthopedic surgery involving one lower limb	8 mg of 0.5% hyperbaric bupivacaine		L3-4
Aliye Esmaoglu 2004[26]	1.USA(35); 2.BSA (35)	Elective outpatient knee arthroscopy	1.5 ml (7.5 mg) 0.5% hyperbaric bupivacaine	3 ml (15 mg) 0.5% hyperbaric bupivacaine	L3-4
Manassero 2014[27]	1.USA (40); 2.BSA (40)	Day-case unilateral open inguinal hernia repair	50 mg hyperbaric 2% prilocaine		L2-3
B.B. Osinaike 2007[28]	1.USA (37); 2.BSA (37)	elective unilateral lower limb surgery	10mg (2mls) of 0.5% hyperbaric bupivacaine		L3-4
Debarati Das 2020[7]	1.USA (36); 2.BSA (36)	Undergoing hemiarthroplasty	0.5% hyperbaric bupivacaine 7.5 mg (1.5 mL) and fentanyl 25 µg (0.5 mL)		L3–4 or L4–5
Ewa Karpel 2009[5]	1.USA (27); 2.BSA (27)	Elective unilateral surgery	0.5% hyperbaric bupivacaine (1.2 mL + 0.1 mL per every 10 cm over 170 cm height)	0.5% hyperbaric bupivacaine (2.4 mL + 0.2 mL per every 10 cm over 170 cm height)	L3–4
F. Cicekci 2014[8]	1.USA(20); 2.BSA (20)	Elective inguinal regional surgery	0.5 % hyperbaric bupivacaine 3 ml (15 mg) + morphine 0.2 ml (0.1 mg)		L4-5
G. Fanelli 2000[29]	1.USA (50); 2.BSA (50)	Outpatient knee arthroscopy	8 mg hyperbaric bupivacaine 0.5%		L3-4
Li Zhu 2014[9]	1.USA (30); 2.BSA (30)	Total Hip Replacement	3ml of hypobaric 0.25% bupivacaine (7.5mg)	2.4ml hypobaric bupivacaine 0.5% (12mg)	L3-4
Lona S. 2022[30]	1.USA (45); 2.BSA (45)	Lower leg surgery	Hyperbaric Bupivacaine 7.5mg	Hyperbaric Bupivacaine 12.5mg	L3-4
Musabaev A. 2012[31]	1.USA(25); 2.BSA(25) 1.USA (25); 2.BSA (25)	Percutaneous nephrolithotomy nephrolithotomy Percutaneous nephrolithotomy	0.05 mg/cm of height hyperbaric bupivacaine	12-18mg hyperbaric bupivacaine	Not reported
M. Yun 2008[32]	1.USA (21); 2.BSA (21)	Elective lower extremity surgery	9 mg of bupivacaine at 5 mg/ml (heavy 0.5% bupivacaine)		L3–4 or L4–5
Nadeem M. 2017[11]	1.USA (30); 2.BSA (30)	Infraumbilical surgeries	2 ml (15 mg) of 0.75% hyperbaric bupivacaine		L3–4 or L4–5
Pakize Kirdemir 2005[13]	1.USA (30); 2.BSA (30)	Elective lower extremity orthopedic surgery	10 mg 0.5% hyperbaric bupivacaine		L4–5
Seyyed M. 2013[33]	1.USA (36); 2.BSA (36)	Lower-limb orthopedic surgery	1.5 mL of hyperbaric bupivacaine 0.5%	2.5 mL of hyperbaric bupivacaine0.5%	L3-4
Sonja S. 2019[34]	1.USA (20); 2.BSA (20)	Unilateral lower limb surgery	7.5mg hyperbaric 0.5% levobupivacaine	15 mg isobaric 0.5% levobupivacaine	L3-4
V. Nesek 2011[35]	1.USA (20);2.BSA (20)	Varicose vein surgery	Hyperbaric levobupivacaine mixed with fentanyl 50 μg	3 ml isobaric 0.5% levobupivacaine (15 mg)	L3-4
Wolfgang G. 2009[36]	1.USA (20); 2.BSA (20)	Elective knee arthroscopy	6 mg hyperbaric 0.5% bupivacaine		L3-4

### Quality assessment

**Figure 2.**
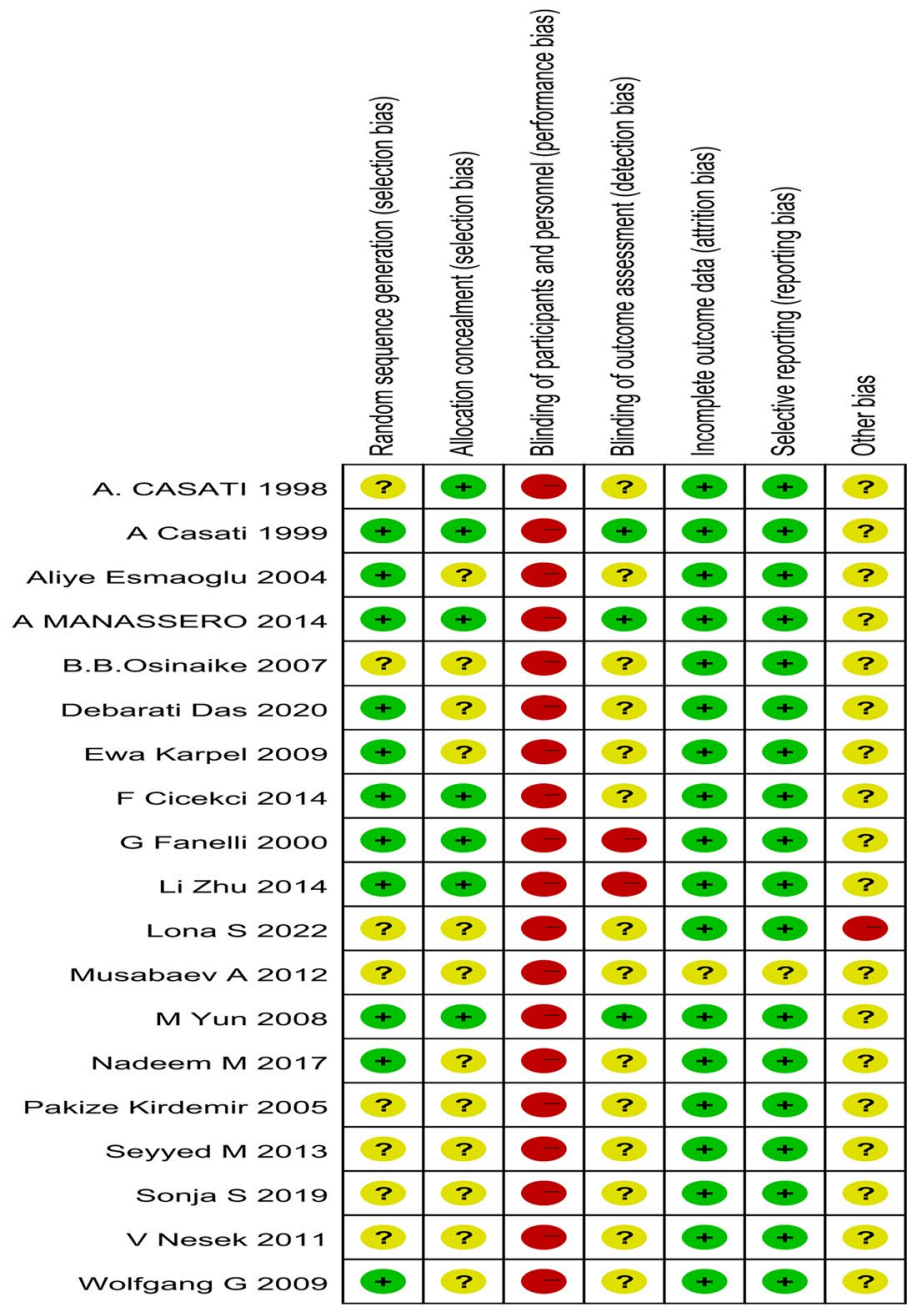
Risk of bias summary.

Risk of bias assessments for the included studies is presented in Figure 2. Only 11 studies described their methods of randomization, and 7 studies described their methods of allocation concealment. All studies had a high risk of bias for blinding of participants and personnel due to the inherent impossibility of blinding when comparing unilateral and bilateral spinal anesthesia. Blinding of outcome assessment was rated as low risk in three studies. Additionally, eighteen studies were deemed to have adequately reported all predefined outcomes. However, low publication quality in some studies contributed to potential bias [30]. As a result, the overall risk of bias in the included trials was assessed as high.

### Primary outcomes

#### Onset time of anesthesia

**Figure 3.**
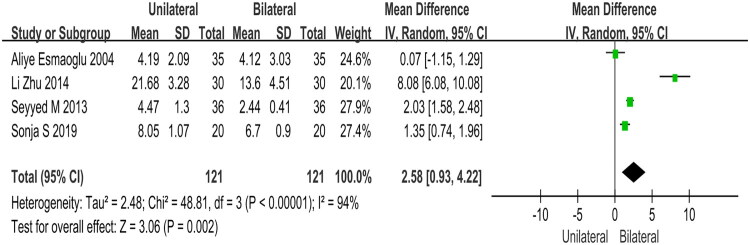
Forest plots of onset time of sensory block.

Four studies [9,26,33,34], including 242 participants, reported on the onset time of sensory block. Meta-analysis suggested that unilateral spinal anesthesia resulted in a longer onset of sensory block compared to bilateral spinal anesthesia (MD = 2.58, 95% *CI*: 0.93–4.22; *p* = 0.002, I^2^ = 94%) (Figure 3). Sensitivity analysis confirmed the stability of this result, though heterogeneity remained high. Due to the limited number of studies, subgroup analyses were not feasible. The quality of evidence was rated as ‘low’ owing to unclear allocation concealment and a high risk of bias related to blinding (Supplementary Data Table 1).

**Figure 4.**
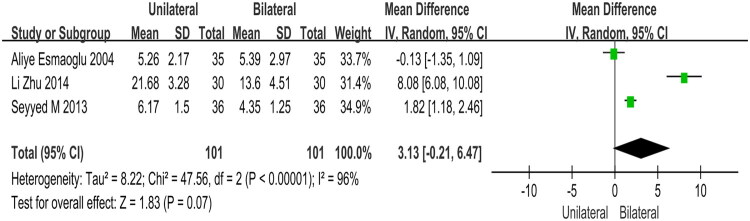
Forest plots of onset time of motor block.

Three studies [9,26,33], involving 202 participants, reported on the onset time of motor block. Analysis suggested that the onset of motor block was longer in unilateral spinal anesthesia than in bilateral spinal anesthesia (MD = 3.13, 95% *CI*: −0.21 to 6.74; *p* = 0.07, I^2^ = 96%) (Figure 4), although the difference was not statistically significant. Sensitivity analysis confirmed the stability of the pooled result, but high heterogeneity persisted. Subgroup analyses were not conducted because of the small number of studies reporting motor block onset times. The quality of evidence was graded as ‘low’ due to unclear allocation concealment and a high risk of bias related to blinding (Supplementary Data Table 1).

#### Duration of anesthesia

**Figure 5.**
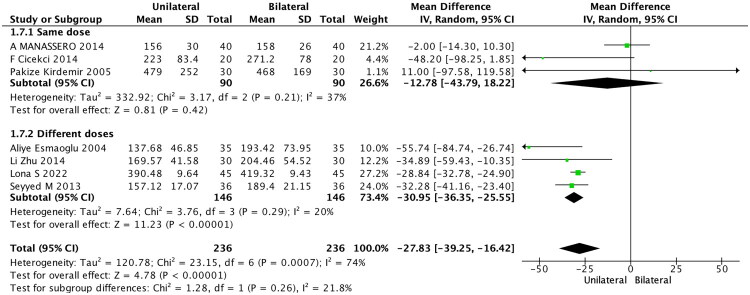
Forest plots of duration of sensory block.

Seven studies [8,9,13,26,27,30,33] involving 472 participants reported the duration of sensory blockade, and analysis showed that the duration of sensory blockade was shorter with unilateral spinal anesthesia than with bilateral spinal anesthesia (MD = −27.83, 95% *CI*: −39.25 to −16.42, *p* < 0.00001, I^2^ = 74%) (Figure 5). However, subsequent sensitivity analyses determined that the pooled analysis result remained stable, despite the heterogeneity remaining high. Dose-based subgroup analyses showed that the duration of sensory blockade was shorter in unilateral spinal anesthesia than in bilateral spinal anesthesia when the dose of local anesthetic used in unilateral anesthesia was less than in bilateral anesthesia. In contrast, when the dose of local anesthetic used was the same for unilateral and bilateral anesthesia, the difference was not statistically significant (Figure 5). The quality of evidence was graded as ‘low’ due to the unclear allocation concealment and high risk of blinding (Supplementary Data Table 1).

**Figure 6.**
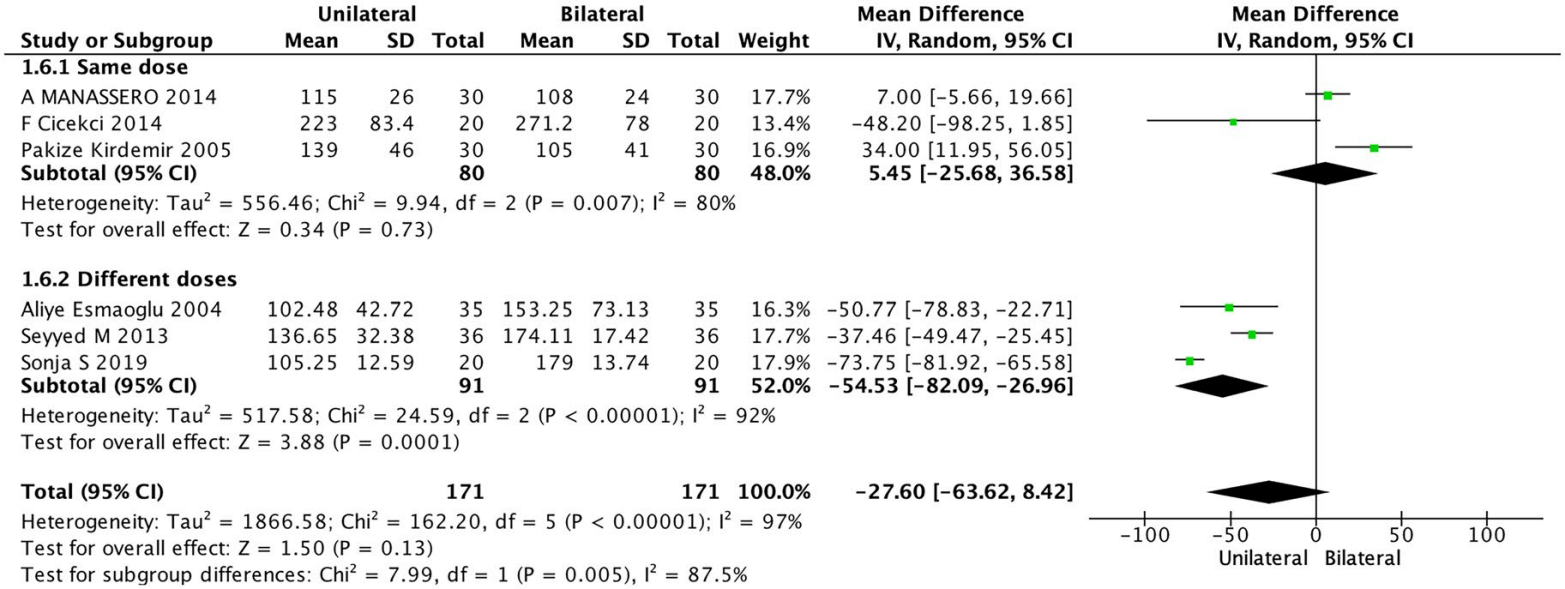
Forest plots of duration of motor block.

Six studies [8,13,26,27,33,34] involving 342 participants reported the duration of motor block, and analysis showed that the duration of motor block was shorter in unilateral spinal anesthesia than in bilateral spinal anesthesia (MD = −27.60, 95% *CI:* −63.62 to 8.42, *p* = 0.13, I^2^ = 97%) (Figure 6). However, this difference was not statistically significant. Subsequent sensitivity analyses determined that the pooled analysis result remained stable, although the heterogeneity remained high. Dose-based subgroup analyses showed that when the dose of local anesthetic used for unilateral anesthesia was less than for bilateral anesthesia, the duration of motor block was shorter in unilateral spinal anesthesia than in bilateral spinal anesthesia. Conversely, the difference was not statistically significant when the same dose of local anesthetic was used for unilateral anesthesia as for bilateral anesthesia (Figure 6). The quality of evidence was graded as ‘low’ due to the unclear allocation concealment and high risk of blinding (Supplementary Data Table 1).

### Secondary outcomes

#### Hypotension

**Figure 7.**
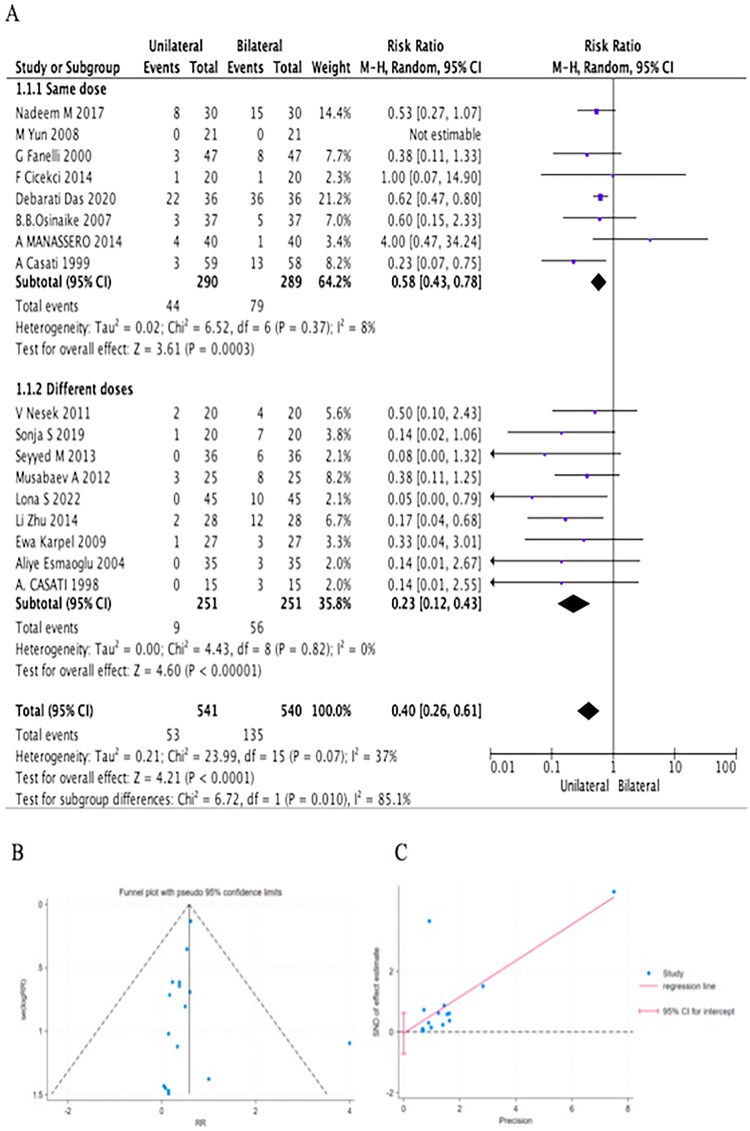
(A) Forest plots of incidence of hypotension. (B) Funnel plot of incidence of hypotension. (C) Assessment of publication bias for hypotension incidence using Egger’s test.

Seventeen studies [5–9,11,25–35] involving 1081 participants reported the incidence of hypotension, and analysis demonstrated that unilateral spinal anesthesia was associated with a significantly lower incidence of hypotension compared with bilateral spinal anesthesia (RR = 0.40, 95% *CI*: 0.31–0.52, *p* < 0.0001, I^2^ = 37%) (Figure 7A). Subgroup analyses stratified by local anesthetic dose showed that this reduction remained statistically significant both when the dose used for unilateral anesthesia was lower than that used for bilateral anesthesia and when equivalent doses were administered. Notably, Egger’s test (*p* = 0.886) and the funnel plot did not reveal significant publication bias (Figure 7B,C). The quality of evidence was graded as ‘Moderate’ due to unclear allocation concealment and high risk of blinding, despite the observed large effect size (Supplementary Data Table 1).

#### Bradycardia

**Figure 8.**
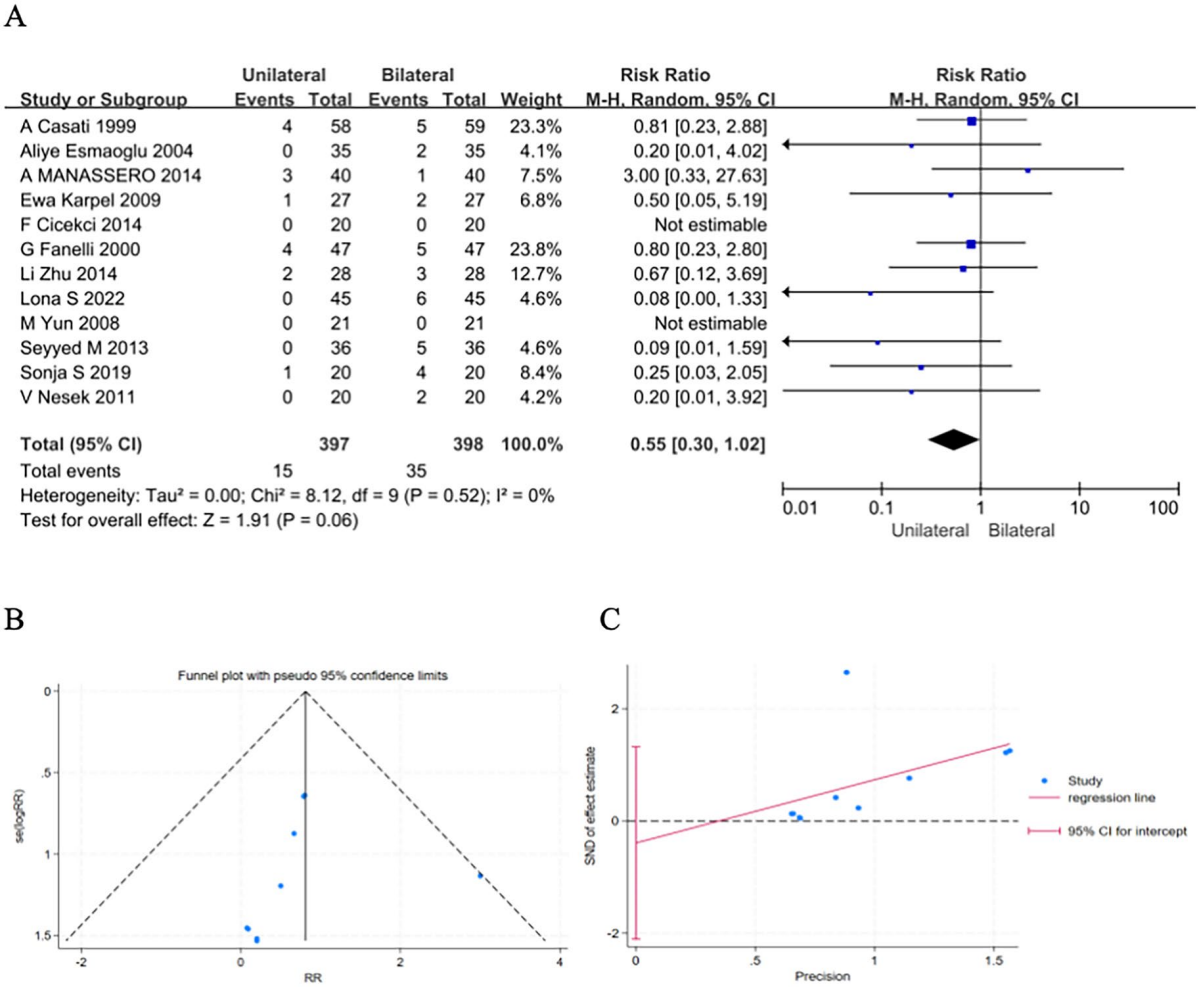
(A) Forest plots of incidence of bradycardia. (B) Funnel plot of incidence of bradycardia. (C) Assessment of publication bias for bradycardia incidence using Egger’s test.

Twelve studies [5,8,9,25–27,29,30,32–35] involving 795 participants reported the incidence of bradycardia. In a combined analysis, although the incidence of bradycardia was lower in unilateral spinal anesthesia than in bilateral spinal anesthesia, the difference did not reach statistical significance (RR = 0.55, 95% CI:0.30–1.02, *p* = 0.06, I^2^ = 0%) (Figure 8A). Egger’s test (*p* = 0.613) and the funnel plot did not reveal significant publication bias (Figure 8B,C). The quality of evidence was graded as ‘low’ due to unclear allocation concealment and high risk of blinding (Supplementary Data Table 1).

#### Urine retention

**Figure 9.**
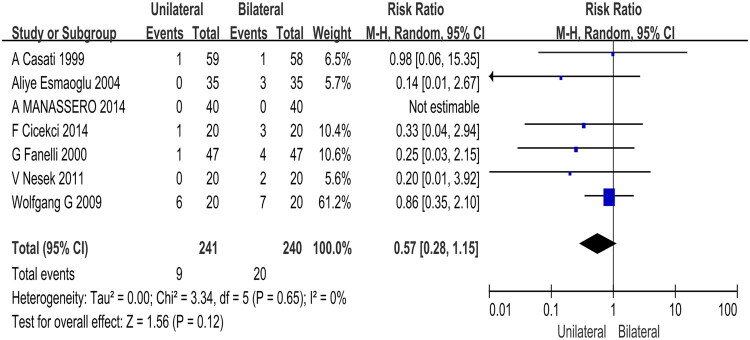
Forest plots of incidence of urinary retention.

Seven studies [8,25–27,29,35,36] involving 481 participants reported the incidence of urinary retention. In a combined analysis, the incidence of urinary retention was lower in unilateral spinal anesthesia than in bilateral spinal anesthesia, but this difference was not statistically significant (RR = 0.57, 95% *CI*: 0.28–1.15, *p* = 0.12, I^2^ = 0%) (Figure 9). The evidence quality was graded as ‘moderate’ due to unclear allocation concealment and a high risk of bias from blinding, despite the observed large effect size (Supplementary Data Table 1).

#### Nausea and vomiting

**Figure 10.**
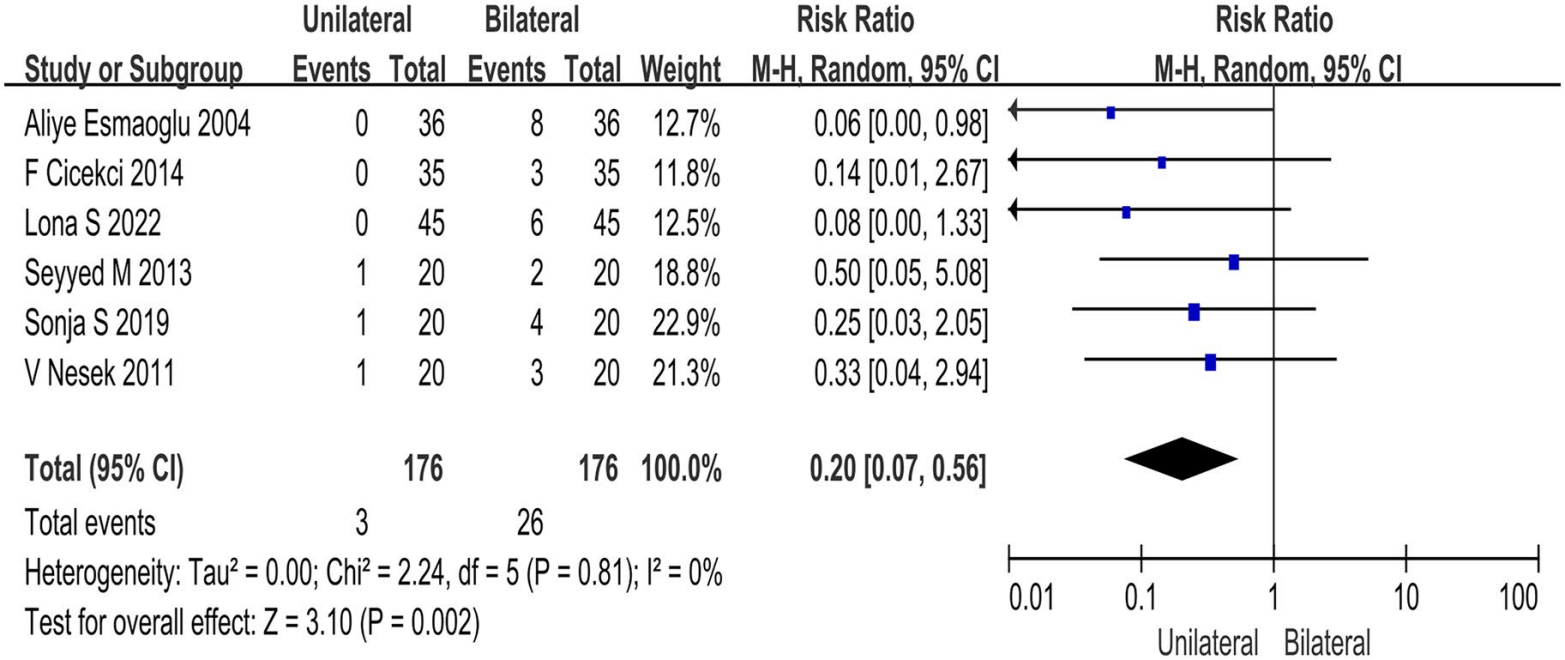
Forest plots of incidence of nausea and vomiting.

Six studies [8,26,30,33–35] involving 352 participants reported the incidence of nausea and vomiting. In a combined analysis, the incidence of nausea and vomiting was lower in unilateral spinal anesthesia than in bilateral spinal anesthesia (RR = 0.20, 95% *CI*: 0.07–0.56, *p* = 0.002, I^2^ = 0%) (Figure 10). The quality of evidence was graded as ‘moderate’ due to unclear allocation concealment and a high risk of bias from blinding, despite the observed large effect size (Supplementary Data Table 1).

#### Postdural puncture headache

**Figure 11.**
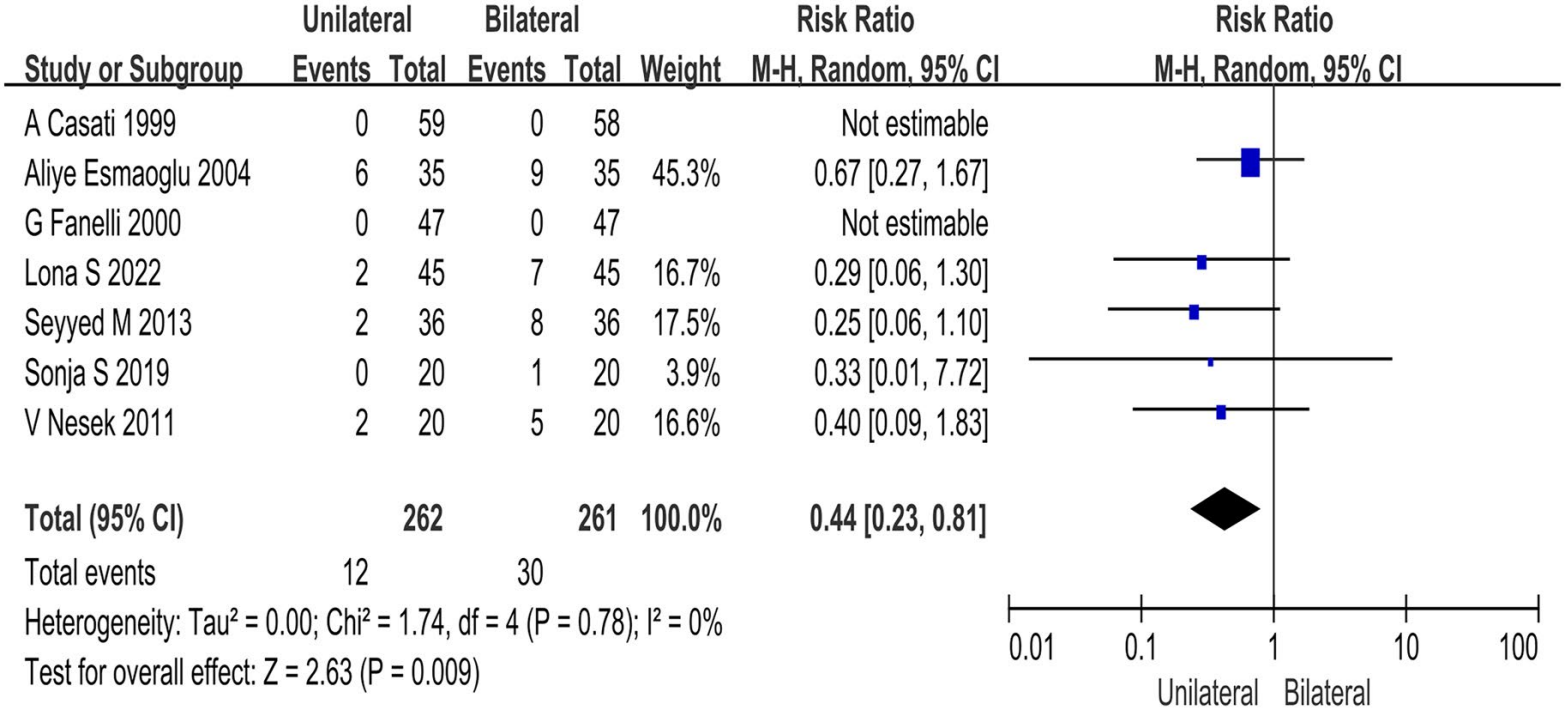
Forest plots of incidence of postdural puncture headache.

Seven studies [25,26,29,30,33–35] involving 523 participants reported the incidence of postdural puncture headache. In a combined analysis, the incidence of headache was lower in unilateral spinal anesthesia than in bilateral spinal anesthesia (RR = 0.44, 95% *CI*: 0.23–0.81, *p* = 0.009, I^2^ = 0%) (Figure 11). The quality of evidence was graded as ‘low’ due to unclear allocation concealment and high risk of blinding (Supplementary Data Table 1).

## Discussion

This systematic meta-analysis demonstrates that bilateral spinal anesthesia is associated with a shorter onset and longer duration of sensory blockade compared to unilateral spinal anesthesia. However, it is also associated with a higher incidence of adverse effects, such as hypotension, nausea, vomiting, and postdural puncture headache. Collectively, these findings suggest that unilateral spinal anesthesia prioritizes hemodynamic stability and safety, whereas bilateral spinal anesthesia achieves more rapid and sustained blockade at the cost of increased complication rates.

Importantly, the observed 2.6-minute prolongation in sensory onset with unilateral spinal anesthesia, although statistically significant, is unlikely to be clinically meaningful in routine practice. In most elective infraumbilical procedures, a delay of fewer than three minutes is unlikely to affect operating room efficiency or patient comfort and therefore probably falls below a minimal clinically important difference threshold. In contrast, the approximately 28-minute reduction in sensory block duration with unilateral anesthesia may carry greater clinical relevance. For short ambulatory procedures, earlier sensory regression may facilitate faster recovery, earlier mobilization, and improved discharge efficiency. Conversely, in longer operations, a shorter block duration may increase the likelihood of intraoperative supplementation or premature regression. Thus, differences in block duration should be interpreted in the context of procedural length.

The differences in onset and duration are likely multifactorial. The pharmacodynamic profile of spinal anesthesia is known to depend on local anesthetic type, dose, injection characteristics, and the use of intrathecal adjuvants [37,38]. Our dose-based subgroup analyses provide important mechanistic insight. When the dose administered for unilateral spinal anesthesia was lower than that used for bilateral spinal anesthesia, unilateral anesthesia was associated with a shorter duration of sensory block. However, when equivalent doses were used, this difference was no longer statistically significant. These findings strongly implicate local anesthetic dose as a principal determinant of the observed differences in block duration. Because only three included studies used intrathecal adjuvants, subgroup analysis based on adjunct use was not feasible. From a clinical perspective, unilateral spinal anesthesia may therefore be particularly suitable for short-duration outpatient procedures, whereas bilateral techniques may be preferable for prolonged or extensive surgeries requiring sustained sensory blockade.

Hemodynamic stability is a central concern in spinal anesthesia. Hypotension, typically occurring in 15%–33% of cases [11], results primarily from sympathetic blockade leading to reduced systemic vascular resistance and cardiac output [39]. In our analysis, unilateral spinal anesthesia was associated with a significantly lower incidence of hypotension compared with bilateral spinal anesthesia, although rates of bradycardia did not differ significantly. The asymmetric distribution of local anesthetic in unilateral techniques may limit the extent of sympathetic blockade, thereby attenuating systemic vasodilation [10]. Notably, dose-based subgroup analyses showed that the reduction in hypotension with unilateral spinal anesthesia remained statistically significant both when a lower dose was used and when equivalent doses were administered in the unilateral group. This finding indicates that the hemodynamic advantage of unilateral spinal anesthesia cannot be explained solely by dose reduction. Other procedural and pharmacological factors—such as the use of intrathecal adjuvants, duration of lateral positioning, and injection rate—may also contribute. Taken together, these results suggest that unilateral spinal anesthesia may confer particular benefit in patients with limited physiological reserve, including elderly individuals and those with compromised cardiovascular function.

Other adverse effects followed a similar trend. Urinary retention, caused by blockade of sacral segments involved in the micturition reflex, typically resolves as sensory function above S3 recovers [40]. Although unilateral spinal anesthesia was associated with a lower incidence of urinary retention, this difference did not reach statistical significance. Nevertheless, the shorter block duration or smaller block extent with unilateral techniques may plausibly reduce the duration of bladder dysfunction, adequately powered trials are required to confirm this possibility.

Likewise, nausea and vomiting occurred less frequently with unilateral spinal anesthesia. These symptoms are closely linked to spinal anesthesia–induced hypotension and autonomic imbalance: (1) Spinal anesthesia-induced sympathetic nerve blockade, leading to vasodilation, hypotension, and reduced cerebral perfusion [41]; (2) Enhanced parasympathetic activity due to sympathetic suppression, increasing gastrointestinal motility [42]. Therefore, the lower incidence of hypotension observed with unilateral techniques may account for the reduced incidence of nausea and vomiting.

Post–dural puncture headache, typically resulting from cerebrospinal fluid leakage and intracranial hypotension after dural puncture [43], was also less common with unilateral spinal anesthesia. However, this outcome is influenced by multiple procedural factors, including needle type and gauge, puncture technique, number of attempts, and patient-specific characteristics [44–46]. Differences in local anesthetic dosing or needle specifications across the included studies may therefore partially explain the observed discrepancy, and causal inference should be made cautiously.

Despite its strengths, this meta-analysis has limitations. The methodological quality of included trials was generally moderate to low, with frequent shortcomings in blinding and allocation concealment. Substantial heterogeneity in anesthetic type, dosing regimens, and adjunct use complicates interpretation. In addition, inconsistent definitions of sensory and motor block across studies may have introduced measurement bias, and restriction to English-language publications may have resulted in language bias. Although some trials used intrathecal adjuncts that could influence hemodynamic and gastrointestinal outcomes, the limited number of these studies precluded meaningful subgroup analysis.

Future research should prioritize rigorously designed randomized controlled trials with standardized dosing strategies, harmonized outcome definitions, and prespecified analyses of adverse events. Such high-quality evidence will be essential to refine patient selection and to define the optimal clinical role of unilateral spinal anesthesia in contemporary perioperative practice.

## Conclusion

Unilateral spinal anesthesia provides a clinically meaningful reduction in hypotension and related adverse effects, while producing a modest delay in onset and a shorter sensory block duration. The difference in onset time may not be clinically significant, whereas the shorter duration may be advantageous or disadvantageous depending on surgical context. Overall, these findings support unilateral spinal anesthesia as a targeted strategy for appropriately selected patients (Elderly individuals and patients with cardiovascular disease), while reaffirming the role of bilateral techniques in longer or more extensive procedures.

## Supplementary Material

Appendix 1 R.docx

PRISMA_2020_checklist.docx

Supplementary Data Table 1 GRADE assessment.docx

## Data Availability

The original contributions presented in the study are included in the article, further inquiries can be directed to the corresponding author.
