# Supplementary material for: Comparing the efficacy and safety of unilateral versus bilateral spinal anesthesia: a meta-analysis and systematic review
Source: Ann Med. 2026 Jun 18;58(1):2689575. doi: 10.1080/07853890.2026.2689575 (PMC13288713; doi:10.1080/07853890.2026.2689575)
Supplement: Appendix 1 R.docx [file IANN_A_2689575_SM1579.docx]

| Search number | Query | Sort By | Filters | Search Details | Results | Time |
| --- | --- | --- | --- | --- | --- | --- |
| 7 | (("Anesthesia, Spinal"[Mesh] OR "Subarachnoid block anesthesia" OR "Spinal Anesthesia" OR "Spinal Anesthesias") AND ("unilateral" OR "One-sided" OR "single-sided")) AND (("Anesthesia, Spinal"[Mesh] OR "Subarachnoid block anesthesia" OR "Spinal Anesthesia" OR "Spinal Anesthesias") AND ("bilateral" OR "double-sided" OR "Two-sided" OR "Conventional")) |  | Clinical Trial | (("anesthesia, spinal"[MeSH Terms] OR "Subarachnoid block anesthesia"[All Fields] OR "Spinal Anesthesia"[All Fields] OR "Spinal Anesthesias"[All Fields]) AND ("unilateral"[All Fields] OR "One-sided"[All Fields] OR "single-sided"[All Fields]) AND (("anesthesia, spinal"[MeSH Terms] OR "Subarachnoid block anesthesia"[All Fields] OR "Spinal Anesthesia"[All Fields] OR "Spinal Anesthesias"[All Fields]) AND ("bilateral"[All Fields] OR "double-sided"[All Fields] OR "Two-sided"[All Fields] OR "Conventional"[All Fields]))) AND (clinicaltrial[Filter]) | 31 | 3:26:03 |
| 6 | (("Anesthesia, Spinal"[Mesh] OR "Subarachnoid block anesthesia" OR "Spinal Anesthesia" OR "Spinal Anesthesias") AND ("unilateral" OR "One-sided" OR "single-sided")) AND (("Anesthesia, Spinal"[Mesh] OR "Subarachnoid block anesthesia" OR "Spinal Anesthesia" OR "Spinal Anesthesias") AND ("bilateral" OR "double-sided" OR "Two-sided" OR "Conventional")) |  |  | ("anesthesia, spinal"[MeSH Terms] OR "Subarachnoid block anesthesia"[All Fields] OR "Spinal Anesthesia"[All Fields] OR "Spinal Anesthesias"[All Fields]) AND ("unilateral"[All Fields] OR "One-sided"[All Fields] OR "single-sided"[All Fields]) AND (("anesthesia, spinal"[MeSH Terms] OR "Subarachnoid block anesthesia"[All Fields] OR "Spinal Anesthesia"[All Fields] OR "Spinal Anesthesias"[All Fields]) AND ("bilateral"[All Fields] OR "double-sided"[All Fields] OR "Two-sided"[All Fields] OR "Conventional"[All Fields])) | 73 | 3:25:53 |
| 5 | ("Anesthesia, Spinal"[Mesh] OR "Subarachnoid block anesthesia" OR "Spinal Anesthesia" OR "Spinal Anesthesias") AND ("bilateral" OR "double-sided" OR "Two-sided" OR "Conventional") |  |  | ("anesthesia, spinal"[MeSH Terms] OR "Subarachnoid block anesthesia"[All Fields] OR "Spinal Anesthesia"[All Fields] OR "Spinal Anesthesias"[All Fields]) AND ("bilateral"[All Fields] OR "double-sided"[All Fields] OR "Two-sided"[All Fields] OR "Conventional"[All Fields]) | 624 | 3:25:41 |
| 4 | ("Anesthesia, Spinal"[Mesh] OR "Subarachnoid block anesthesia" OR "Spinal Anesthesia" OR "Spinal Anesthesias") AND ("unilateral" OR "One-sided" OR "single-sided") |  |  | ("anesthesia, spinal"[MeSH Terms] OR "Subarachnoid block anesthesia"[All Fields] OR "Spinal Anesthesia"[All Fields] OR "Spinal Anesthesias"[All Fields]) AND ("unilateral"[All Fields] OR "One-sided"[All Fields] OR "single-sided"[All Fields]) | 393 | 3:25:02 |
| 3 | "bilateral" OR "double-sided" OR "Two-sided" OR "Conventional" |  |  | "bilateral"[All Fields] OR "double-sided"[All Fields] OR "Two-sided"[All Fields] OR "Conventional"[All Fields] | 978,920 | 3:24:04 |
| 2 | "unilateral" OR "One-sided" OR "single-sided" |  |  | "unilateral"[All Fields] OR "One-sided"[All Fields] OR "single-sided"[All Fields] | 168,372 | 3:23:46 |
| 1 | "Anesthesia, Spinal"[Mesh] OR "Subarachnoid block anesthesia" OR "Spinal Anesthesia" OR "Spinal Anesthesias" |  |  | "anesthesia, spinal"[MeSH Terms] OR "Subarachnoid block anesthesia"[All Fields] OR "Spinal Anesthesia"[All Fields] OR "Spinal Anesthesias"[All Fields] | 17,423 | 3:23:25 |

Figure 1. Search Strategy for PubMed


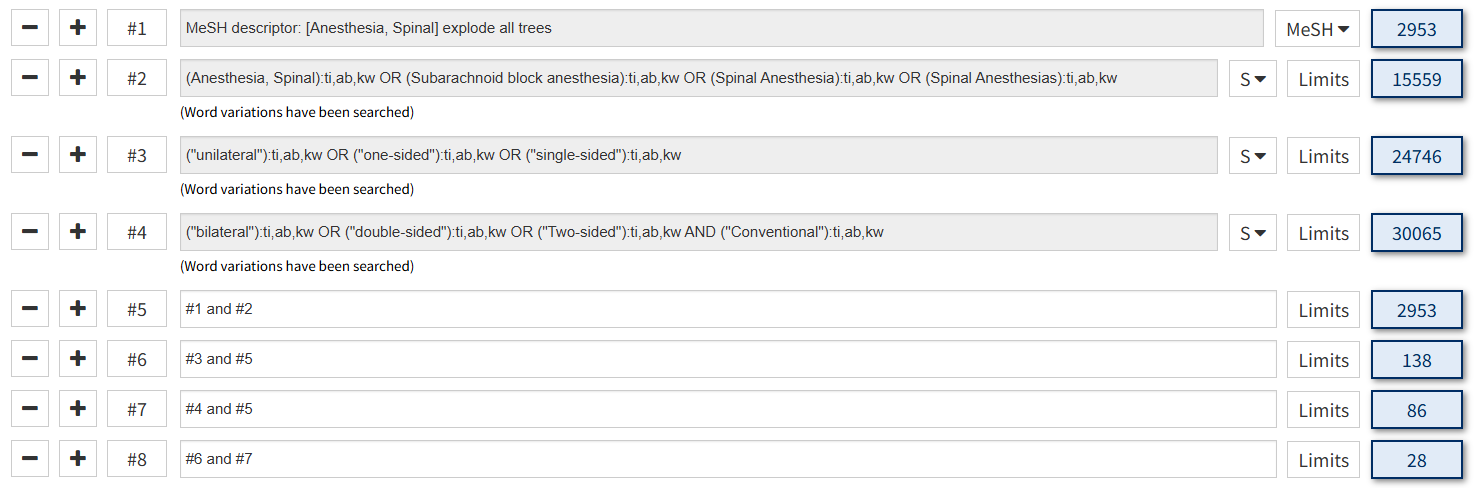


Figure 2. Search Strategy for the Cochrane Library


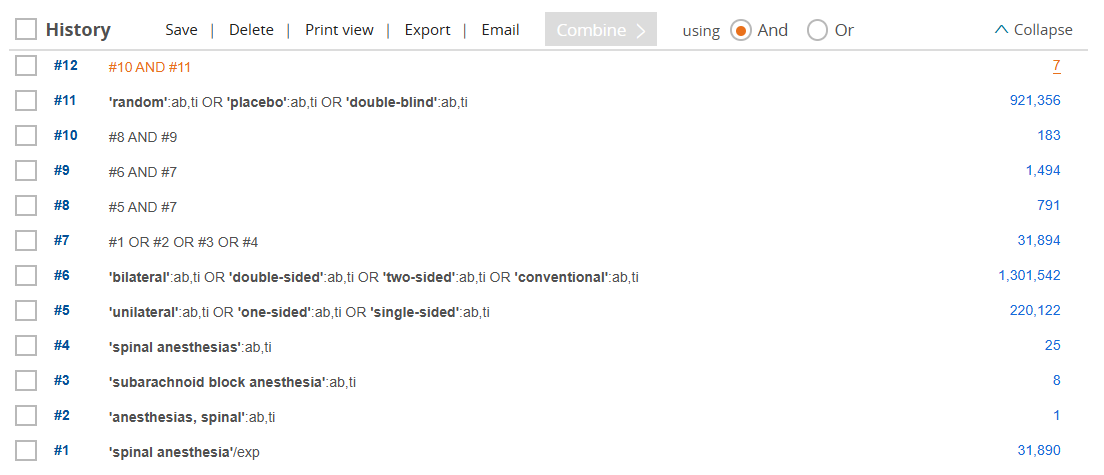


Figure 3. Search Strategy for Embase
