# Supplementary material for: Comparing the efficacy and safety of unilateral versus bilateral spinal anesthesia: a meta-analysis and systematic review
Source: Ann Med. 2026 Jun 18;58(1):2689575. doi: 10.1080/07853890.2026.2689575 (PMC13288713; doi:10.1080/07853890.2026.2689575)
Supplement: Supplementary Data Table 1 GRADE assessment.docx [file IANN_A_2689575_SM1577.docx]

**Author(s):**
**Date:** 2024-12-17
**Question:** Unilateral vs bilateral for Efficacy and Safety
**Settings:**
**Bibliography:** . Unilateral spinal anesthesia versus bilateral spinal anesthesia for Efficacy and Safety. Cochrane Database of Systematic Reviews [Year], Issue [Issue].

| **Quality assessment** | | | | | | | **No of patients** | | **Effect** | | **Quality** | **Importance** |
| --- | --- | --- | --- | --- | --- | --- | --- | --- | --- | --- | --- | --- |
|  |  |  |  |  |  |  |  |  |  |  |  |  |
| **No of studies** | **Design** | **Risk of bias** | **Inconsistency** | **Indirectness** | **Imprecision** | **Other considerations** | **Unilateral** | **Bilateral** | **Relative (95% CI)** | **Absolute** |  |  |
| **Hypotension** | | | | | | | | | | | | |
| 17 | randomised trials | very serious | no serious inconsistency | no serious indirectness | no serious imprecision | strong association | 53/541 (9.8%) | 135/540 (25%) | RR 0.4 (0.31 to 0.52) | 150 fewer per 1000 (from 120 fewer to 172 fewer) | ⊕⊕⊕O  Moderate | IMPORTANT |
|  |  |  |  |  |  |  |  | 20% |  | 120 fewer per 1000 (from 96 fewer to 138 fewer) |  |  |
| **Headache** | | | | | | | | | | | | |
| 7 | randomised trials | very serious^1,2^ | no serious inconsistency | no serious indirectness | no serious imprecision | none | 12/262 (4.6%) | 30/261 (11.5%) | RR 0.44 (0.23 to 0.81) | 64 fewer per 1000 (from 22 fewer to 89 fewer) | ⊕⊕OO  LOW | IMPORTANT |
|  |  |  |  |  |  |  |  | 15.6% |  | 87 fewer per 1000 (from 30 fewer to 120 fewer) |  |  |
| **Bradycardia** | | | | | | | | | | | | |
| 12 | randomised trials | very serious^1,2^ | no serious inconsistency | no serious indirectness | no serious imprecision | none | 15/397 (3.8%) | 35/398 (8.8%) | RR 0.46 (0.26 to 0.8) | 47 fewer per 1000 (from 18 fewer to 65 fewer) | ⊕⊕OO  LOW | IMPORTANT |
|  |  |  |  |  |  |  |  | 9.2% |  | 50 fewer per 1000 (from 18 fewer to 68 fewer) |  |  |
| **Urine retention** | | | | | | | | | | | | |
| 7 | randomised trials | very serious^1,2^ | no serious inconsistency | no serious indirectness | no serious imprecision | strong association | 9/241 (3.7%) | 20/240 (8.3%) | RR 0.57 (0.28 to 1.15) | 36 fewer per 1000 (from 60 fewer to 12 more) | ⊕⊕⊕O  Moderate | IMPORTANT |
|  |  |  |  |  |  |  |  | 8.6% |  | 37 fewer per 1000 (from 62 fewer to 13 more) |  |  |
| **Nausea and vomiting** | | | | | | | | | | | | |
| 6 | randomised trials | very serious^1,2^ | no serious inconsistency | no serious indirectness | no serious imprecision | strong association | 3/176 (1.7%) | 26/176 (14.8%) | RR 0.2 (0.07 to 0.56) | 118 fewer per 1000 (from 65 fewer to 137 fewer) | ⊕⊕⊕O  Moderate | IMPORTANT |
|  |  |  |  |  |  |  |  | 14.2% |  | 114 fewer per 1000 (from 62 fewer to 132 fewer) |  |  |
| **Onset time of sensory block (Better indicated by lower values)** | | | | | | | | | | | | |
| 4 | randomised trials | very serious^1,2^ | no serious inconsistency | no serious indirectness | no serious imprecision | none | 121 | 121 | - | MD 2.58 higher (0.93 to 4.22 higher) | ⊕⊕OO  LOW | CRITICAL |
| **Onset time of motor block (Better indicated by lower values)** | | | | | | | | | | | | |
| 3 | randomised trials | very serious^1,2^ | no serious inconsistency | no serious indirectness | no serious imprecision | none | 101 | 101 | - | MD 3.13 higher (0.21 lower to 6.47 higher) | ⊕⊕OO  LOW | CRITICAL |
| **Duration of sensory block (Better indicated by lower values)** | | | | | | | | | | | | |
| 7 | randomised trials | very serious^1,2^ | no serious inconsistency | no serious indirectness | no serious imprecision | none | 236 | 236 | - | MD 24.03 lower (39.77 to 8.29 lower) | ⊕⊕OO  LOW | CRITICAL |
| **Duration of motor block (Better indicated by lower values)** | | | | | | | | | | | | |
| 6 | randomised trials | very serious^1,2^ | no serious inconsistency | no serious indirectness | no serious imprecision | none | 171 | 171 | - | MD 27.6 lower (63.62 lower to 8.42 higher) | ⊕⊕OO  LOW | CRITICAL |

^1^ High risk of Blinding of participants and personnel
^2^ Unclear risk of concealment
